# Cutaneous disorders of adolescence among Nigerian secondary school students

**DOI:** 10.11604/pamj.2020.36.36.21089

**Published:** 2020-05-27

**Authors:** Jadesola Tryphena Oyedepo, Oludolapo Sherifat Katibi, Olanrewaju Timothy Adedoyin

**Affiliations:** 1 Department of Paediatrics, University of Ilorin, Ilorin, Nigeria; 2 South Shore Women's and Children's Hospital, Victoria Island, Lagos, Nigeria

**Keywords:** Skin, adolescence, community

## Abstract

**Introduction:**

A community-based, age-specific survey of skin disorders is usually necessary to characterize the true burden of skin disease among a given population and help to tailor health care personnel training and delivery towards the prevalent disorders in resource poor settings.

**Methods:**

This was a descriptive cross-sectional study among adolescents attending secondary schools in Ilorin, Kwara State, Nigeria. A thousand and three hundred students were recruited from public and private secondary schools through a multi-staged stratified random sampling method. Information was obtained via a semi-structured questionnaire and all students underwent a physical examination. Data was analysed using SPSS version 20. Information generated was presented with tables and figures.

**Results:**

The prevalence of skin disease in the study was 66.5%. More females, mid-adolescents, students in senior class and those attending public schools had skin disorders. The most prevalent skin disease were: acne vulgaris, pityriasis versicolor, tinea capitis, pityriasis capitis and traction alopecia.

**Conclusion:**

Skin conditions are highly prevalent among the adolescent population. Infective and inflammatory skin conditions appear to be more prevalent than other classes. Most times, only a few skin disorders account for the bulk of dermatoses affecting this age group. Adolescent skin healthcare should be subsidized because of the high prevalence of skin disorders in this age group.

## Introduction

Dermatological disorders are common all over the world, affecting people of all races and age groups. Skin disorders have been estimated to affect between 30% and 70% of individuals worldwide, with even higher rates in at-risk subpopulations [1]. In 2010, the global burden of disease study of 187 countries determined that, all over the world, skin conditions were the fourth leading cause of non-fatal disease burden and collectively, they ranged from the 2^nd^ to the 11^th^ cause of years lived, with a disability at the country level [2]. Akinkugbe *et al.* [3] in 2016 found a prevalence rate for skin disorders of 19.2% in adults and 25.2% in children in a rural community in Lagos, Nigeria, highlighting the high burden of skin disorders in the paediatric population. The World Health Organization (WHO) defines an adolescent as an individual between the age of 10 and 19 years [4]. About 1 in 6 persons in the world is an adolescent, making about 1.2 billion people [5].

Adolescents make up approximately 38 million of the population of Nigeria, a number that exceeds the total population of some countries [6, 7]. Puberty, which is a landmark event during adolescence, is the biologic transition from childhood to adulthood and is characterised by profound hormonal influences that effects changes in many organs of the body including the skin [8]. The adolescent years are formative periods in the life of every child where the foundation for future health, particularly the mental and social domains is formed. Changes in the composition of the skin and its appendages are quite prominent during this phase, placing them at risk of certain types of dermatologic problems [9, 10]. Skin disorders in these age group can have far reaching consequences because a lot of the adolescent's self-esteem is tied to his/her body image [11]. There is, however, a dearth of studies addressing skin disorders in the adolescent age group as most studies in this environment was exclusive to children or included only young adolescents. Identifying the peculiar dermatological needs of this population therefore becomes imperative. This study aimed to determine the prevalence and spectrum of cutaneous disorders among adolescent children, to find the associations if any between the occurrence of skin disorders and some socio-demographic indices and to evaluate the skin health-seeking behavior of adolescents in Ilorin, Kwara State.

## Methods

This was a descriptive cross-sectional study that was conducted in 16 secondary schools in Ilorin, Kwara State, Nigeria. Research was approved by the University of Ilorin Teaching Hospital Ethical Review Board with Ethical approval number ERC PAN 2017/03/1655. Additional approval was obtained from the Kwara State Ministry of Education. The study was carried out by 2 paediatric dermatologists with the assistance of paediatric dermatology trainees and research assistants. Sixteen schools out of the 145 secondary schools in the Ilorin metropolis were selected through a multi-staged stratified random sampling method.

Study participants were recruited over a 5-month period from November 2017 to March 2018. The research team paid a first visit to the selected schools during which the school management was informed about the study and their consent sought. A second visit was made to the selected schools a week before the research commenced and students selected by simple random sampling were addressed and given detailed information about the research. They were given assent forms to sign and consent forms to take home to their parents. Only those who returned with a duly filled and signed assent and consent form were included in the study.

All study participants were given semi-structured questionnaires which was administered by the researchers. Information related to socio-demographics, presence and history of skin disorder, were obtained. The social class of the subjects was determined using the Oyedeji's classification system (Social class I&II: Upper, social class III: Middle and social class IV&V: Lower) [12]. Examination was done in private, well-lit rooms in the presence of chaperones. Diagnosis was mainly clinical and where necessary, dermoscopy and microscopy was done. Disorders requiring further evaluation and treatment were referred to the paediatric dermatology clinic of the University of Ilorin Teaching Hospital while those with readily treatable conditions were given appropriate prescriptions. Clinical photograph of skin lesions was also taken. Data was entered and analyzed using the statistical package for social sciences (Version 20). Frequency tables and charts were generated and differences between proportions of categorical variables were tested using the chi square test. The confidence interval was set at 95% and a p-value less than 0.05 was considered statistically significant.

## Results

**Socio-demographic characteristics of study population:** a total of 1300 students were recruited from public and private secondary schools in Ilorin. The mean age of the study population was 13.8±2.1 years with an age range of 10-19 years. Male: Female ratio was 1:1.3.

**Figure 1:**
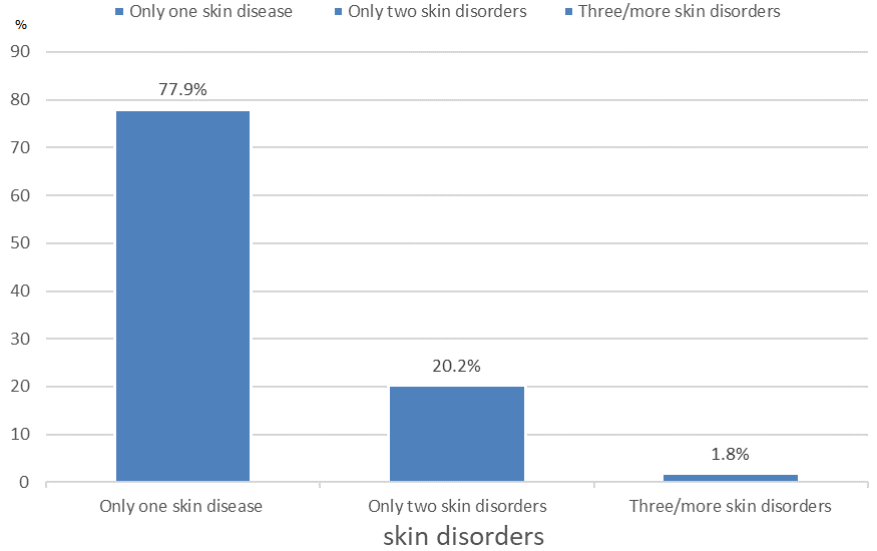
Number of skin disorders among the students with dermatological diseases

**Prevalence of skin disorders in the study population:** eight hundred and sixty-five students had at least one skin disorder following a physical examination, giving a prevalence of 66.5%. There was a higher prevalence of self-reported skin disorders with 951 (73.1%) students reporting a skin complaint compared to 865 (66.5%) who were determined to have dermatologic diseases following history and physical examination. Most students (77.9%) had one skin disease while 20.2% had 2 skin disorders and 1.8% had three or more skin disorders as depicted in Figure 1.

**Distribution of skin disorders across socio-demographic indices:** skin disorders were more common in females, students attending public schools, students in the senior secondary classes and mid-adolescents (p<0.05) as shown in Table 1.

**Table 1:** Occurrence of skin disorder and socio-demographic indices

Socio-demographic	Skin disorder present	Skin disorder Absent	χ2*	p value
**Gender**				
Male	349 (42.8)	224 (51.5)	14.592	0.001
Female	516 (63.3)	211 (48.5)		
**Type of school**				
Public	492 (70.9)	202 (29.1)	12.681	0.001
Private	373 (61.6)	233 (38.4)		
**School class**				
Junior	371 (59.6)	252 (40.4)	26.237	0.001
Senior	494 (73.0)	183 (27.0)		
**Age group**				
Early adolescent	354 (60.3)	233 (39.7)	22.846	0.001
Mid adolescent	393 (69.8)	170 (30.2)		
Late adolescent	118 (78.7)	32 (21.3)		

* Χ2 = Chi-square

**Spectrum of skin disorders among adolescents:** a total of 48 diagnoses was made. Skin disorders affecting the skin appendages 428 (49.5%) were the most frequent followed by infective skin disorders 330 (38.2%). The top five diagnoses were acne vulgaris 347(40.1%), pityriasis versicolor 204 (23.6%), tinea capitis 80 (9.1%) pityriasis capitis 77 (8.9%) and traction alopecia 64 (7.4%) (Table 2).

**Table 2:** Spectrum of skin disorders in the study population

Disorders of appendages	463 (43.2%)	Infections	356 (33.2%)	Dermatitis	93 (8.7%)
Acne vulgaris	347 (40.1)	Pityriasis versicolor	204 (23.6)	Pityriasis capitis	77 (8.9)
Traction alopecia	64(7.4)	Tinea capitis	80 (9.1)	Atopic dermatitis	8 (0.9)
Traction folliculitis	15 (1.7)	Tinea pedis	22 (2.5)	Seborrheic dermatitis	4 (0.5)
Hyperhidrosis	9(1.0)	Tinea corporis	20 (2.3)	Irritant dermatitis	2 (0.2)
Milaria	7 (0.8)	Pyodermas	12 (1.3)	Nickel dermatitis	1 (0.1)
Ingrowing toenail	5 (0.6)	Onychomycosis	7 (0.8)	Lichen simplex chronicus	1 (0.1)
Scarring alopecia	4 (0.5)	Pyogenic granuloma	4 (0.5)	**Papulosquamous disorders**	**8 (0.7)**
Hirsutism	3 (0.3)	Paronychia	3 (0.3)	Lichen nitidus	4 (0.5)
Acne keloidalis nuchae	2 (0.2)	Scabies	2 (0.2)	Pityriasis rosea	2 (0.2)
Beau lines	2 (0.2)	Viral warts	1 (0.1)	Lichen planus	1 (0.1)
Dystrophic nails	2 (0.2)	Tinea cruris	1 (0.1)	Lichen striatus	1 (0.1)
canitis	1 (0.1)	**Atrophy/hypertrophy**	**33 (3.1%)**	**Others**	**37 (3.5%)**
Alopecia areata	1 (0.1)	Striae distensae	18 (2.1)	Congenital naevus	24 (3.2)
Pseudofolliculitis barbae	1 (0.1)	Hypertrophic scar	11 (1.3)	Dermatosis papulosa nigra	4 (0.5)
**Urticaria/erythema**	**36 (3.4%)**	Keloids	4 (0.5)	Acanthosis nigricans	3 (0.3)
Papular urticaria	35 (4.0)			Keratoderma	3 (0.3)
Chronic urticaria	1(0.1)			Xerosis	2 (0.2)
**Pigmentary**	**46 (4.3%)**			Granuloma annulare	1 (0.1)
Post-inflammatory dyspigmentation	24(2.8)				
Exogenous ochronosis	19 (2.2				
Albinism	2 (0.2)				
Piebaldism	1 (0.1)				

**Figure 2:**
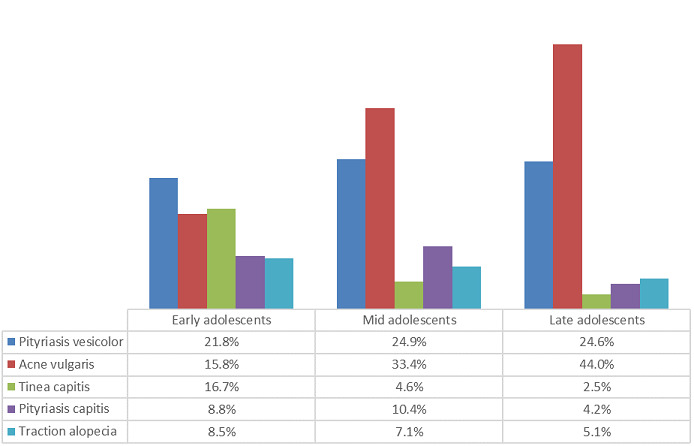
Prevalent skin disorders across age groups

**Occurrence of skin disorders across the different age groups:** pityriasis versicolor was the most prevalent disorder among early adolescents (21.8%), while acne vulgaris was most prevalent in mid and late adolescence (33.4% and 44% respectively). Traction alopecia, tinea capitis and pityriasis capitis were the other common disorders seen. Tinea pedis was more common in late adolescence than tinea capitis (Figure 2).

**Occurrence of skin disorders across gender:** infective dermatoses were more prevalent among the male subjects compared to the females and traction alopecia occurred exclusively in the female population (Table 3).

**Table 3:** Gender distribution of the top five skin disorders

Male	Female
Pityriasis versicolor (33.8%)	Acne vulgaris (31.8%)
Tinea capitis (21.8%)	Pityriasis versicolor (16.0%)
Acne vulgaris (20.2%)	Traction alopecia (12.0%)
Pityriasis capitis (5.7%)	Pityriasis capitis (11.0%)
Tinea corporis (3.7%)	Papular urticaria (4.3%)

**Figure 3:**
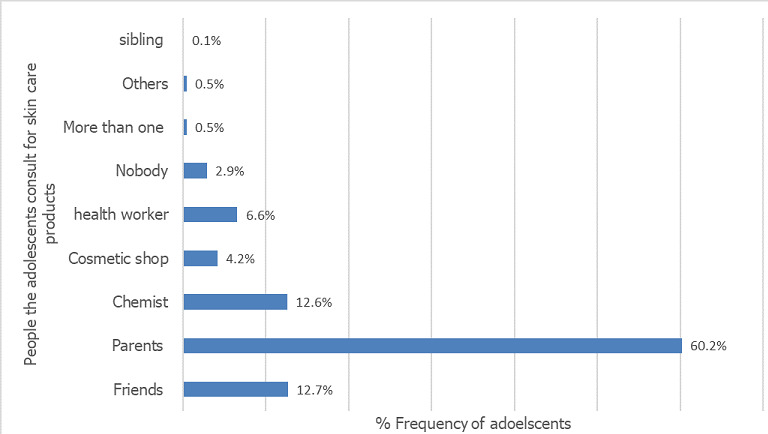
Bar chart showing people the adolescents regularly consult for skin care products

**Health-seeking behavior and skin care consultation practice of adolescents with skin disorders:** of the 865 patients who had skin disorders, only 195 (22.5%) had gone to the hospital on account of the skin complaint, 69.1% had never seen a health worker for skin complaint and 8.3% were not sure if they had. The bar chart in Figure 3 shows the people the adolescents affected by skin disorders consulted for products to use.

## Discussion

**Statement of principal findings:** this study showed a high prevalence of skin disorders among adolescents attending secondary schools in Nigeria with well over half of the study population having at least one skin disorder. This high prevalence is similar to what Ogunbiyi *et al.* [13] and Yaseen *et al.* [14] found among adolescents in Nigeria and India. Using the International Classification of Diseases (ICD) for skin disorders, we found that diseases of the skin appendages were the most frequent subclass of skin disorders seen [15]. This is not unusual as acne, which was the major contributor in this class, is generally considered a disease of adolescence and several studies have corroborated this [16-18]. Oninla *et al.* [19] also found a high prevalence of disorders of the skin appendages attributable to acne vulgaris in a cohort of adolescents in Ile-Ife. Other prevalent diseases were superficial fungal infections - pityriasis versicolor and tinea capitis - which were more prevalent in male students and early adolescents. A similar finding has been previously reported [20, 21]. However, Ibekwe *et al.* [22] found a higher prevalence in female adolescents in some Nigerian secondary schools. Traction alopecia was one of the top five diseases and occurred exclusively in the female population.

A study in South Africa reported similar findings although another study described traction alopecia among Sikh males and this was attributed to cultural practices [23, 24]. The spectrum of skin disease in this study was wide with a total of 48 diagnoses, however only a handful (about five skin disease) affected more than 80% of the study population. Quite a number of the female population had exogenous ochronosis, a side effect of the use of skin lightening creams. Skin lightening is a harmful practice that is fast becoming an epidemic even among younger populations. Two studies, one in Ghana, the other in Nigeria corroborate this [25, 26]. An audit for health seeking behavior with respect to skin complaints also revealed that these students rarely visit the hospitals for skin care despite the high prevalence of skin disease among them. Surprisingly, most of them asked their parents for skin care advice. Health seeking behavior among adolescents is generally poor coupled with the fact that skin disorders are rarely fatal, this may have contributed to the low health seeking behavior seen in this study [27, 28].

**Strength and weakness of the study:** one of the strengths of this study is the large sample size and also the recruitment of the entire range of the adolescent population as defined by the World Health Organization [4]. This allowed for a comprehensive evaluation of the effects of the different stages of adolescence on the skin and a comparison of these effects. Our methodology also ensured a thorough, full body examination that enabled us to identify skin disorders that are easy to miss such as hair and scalp disorders. This study was community-based, targeting the adolescent age group by focusing on schools, where a lot of them are located. This ensured that we could reach a larger adolescent population as secondary school enrollment rate is about 74% in Kwara State [29]. Our study also recruited students attending both private and public secondary schools, allowing for an objective evaluation of disease occurrence which may be attributable to school type. One weakness of our study is that it did not characterize specific skin disease as there was a wide spectrum with up to 48 skin diseases and as such definite characteristics of single skin disorders was not elicited.

**Strength and weakness in relation to other studies:** this study had a large sample size, which is similar to the methodology other dermato-epidemiologic skin surveys done among adolescents in Nigeria employed. A large sample size ensured that the results of this study can be reliably generalized over the adolescent population. Ogunbiyi *et al.* [13] and Henshaw *et al.* [30] also recruited over a thousand adolescents and had similar prevalence rates of 70.8% and 64.2% respectively. However, in Dar es salaam, Komba and associates [31] had a smaller sample size and this might be why they had a lower prevalence rate of 57.3% among the adolescents they recruited. As earlier stated, we recruited students aged between 10 and 19 years as this is the adolescent age group according to the World Health Organization (WHO) [32]. The prevalent disorders in the adolescents in this study were acne vulgaris, pityriasis versicolor, tinea capitis, pityriasis capitis and traction alopecia. In contrast, Popescu *et al.* [33] in Romania who studied only early adolescents reported infective skin disorders as the most common type of dermatoses seen. Similarly, a study among only female adolescents in Hyderabad, Pakistan had hair loss and hirsutism as one of the prominent skin disorders seen [34]. These skin diseases are generally more common among females.

Our study was community based and therefore the prominent disorders reported are different from what is mostly seen in hospital-based surveys. In Lagos Nigeria, Ayanlowo *et al.* [35] found infections, eczematous disorders, infestations, papulosquamous disorders and bullous eruptions were more prevalent in children attending a general dermatology clinic. It is not unusual to find that skin disorders that are prominent in the community are different from those seen in the hospitals. Hospital visits for skin disorders is likely to be influenced by factors such as chronicity of illness, presence of disfigurement and co-morbidities, socio-economic status and exposure [27, 36]. Whereas, what is seen in the community is likely to be more representative of the burden of dermatoses in the society as the bias of health-seeking behavior has been removed. We classified adolescents using the United Nations Children's Fund (UNICEF) classification where early adolescence is from 10 to 13 years, mid adolescence is from 14 to 16 years and late adolescence is from 17 to 19 years [4]. Classifying adolescents helps to determine what skin dermatoses is peculiar to a particular age group and identify reasons why if possible. In a study in Turkey, students were recruited from age 14 years and above which correlates with mid adolescence according to the UNICEF classification, thereby omitting early adolescents [37]. Eshan's study in Calabar Nigeria also recruited from mid adolescence upwards, this makes it difficult to compare study outcomes [30]. We did not explore the individual characteristics of single disease entities in this study and thus cannot make in-depth analysis of individual dermatoses seen. Some skin surveys target single entities and are able to characterize the disease and identify determinants of its occurrence or its effect on other areas of functioning. Ogedegbe *et al.* [38] focused on acne vulgaris and its effect on quality of life, Ibekwe *et al.* [22] also looked at pityriasis versicolor and quality of life. These studies although did not include many other skin diseases that are prevalent in the adolescent age group, were able to make more inferences.

**Meaning of the study:** this study has shown a high burden of skin disease in school going adolescents in a typical Nigerian community. It has also been able to demonstrate that the occurrence of skin disease can be influenced by age and gender even among adolescents. It also evaluated the adolescent behavior with regards to skin and health.

**Unanswered questions and future research:** a study on the determinants of skin disease in adolescence and the effect on quality of life will provide further insight into the burden of these disorders.

## Conclusion

In conclusion, this study has corroborated the fact that the burden of skin disease is heavy in Nigerian adolescents going by similar prevalence rates in studies from some parts of the country. Another important finding is that almost 90% of skin disorders can be accounted for by a handful and as such targeted efforts can be made to train community health workers, subsidize dermatologic care in this age group and create more awareness about skin disorders.

### What is known about this topic

- The prevalence of skin disease is high in adolescents;
- There is a wide spectrum of skin disease that can affect adolescents.

### What this study adds

- The prevalence of traction alopecia is high in female adolescents;
- A few dermatoses account for the bulk of skin disorders seen in adolescents;
- Skin health-seeking behavior is poor among adolescents and is largely influenced by their parents.

## Competing interests

The authors declare no competing interests.
